# Cactus Pear Mucilage (*Opuntia* spp.) as a Novel Functional Biopolymer: Mucilage Extraction, Rheology and Biofilm Development

**DOI:** 10.3390/polym16141993

**Published:** 2024-07-12

**Authors:** Brandon Van Rooyen, Maryna De Wit, Gernot Osthoff, Johan Van Niekerk

**Affiliations:** 1 Department of Sustainable Food Systems and Development, University of the Free State, Bloemfontein 9301, South Africa; 2 Department of Microbiology and Biochemistry, University of the Free State, Bloemfontein 9301, South Africa

**Keywords:** biopolymer, cactus pear mucilage, biofilms, functional properties, rheology, *Opuntia ficus-indica*, mucilage extraction

## Abstract

The investigation of novel, natural polymers has gained considerably more exposure for their desirable, often specific, functional properties. Multiple researchers have explored these biopolymers to determine their potential to address many food processing, packaging and environmental concerns. Mucilage from the cactus pear (*Opuntia ficus-indica*) is one such biopolymer that has been identified as possessing a functional potential that can be used in an attempt to enhance food properties and reduce the usage of non-biodegradable, petroleum-based packaging in the food industry. However, variations in the structural composition of mucilage and the different extraction methods that have been reported by researchers have considerably impacted mucilage’s functional potential. Although not comparable, these factors have been investigated, with a specific focus on mucilage applications. The natural ability of mucilage to bind water, alter the rheology of a food system and develop biofilms are considered the major applications of mucilage’s functional properties. Due to the variations that have been reported in mucilage’s chemical composition, specifically concerning the proportions of uronic acids, mucilage’s rheological and biofilm properties are influenced differently by changes in pH and a cross-linker. Exploring the factors influencing mucilage’s chemical composition, while co-currently discussing mucilage functional applications, will prove valuable when evaluating mucilage’s potential to be considered for future commercial applications. This review article, therefore, discusses and highlights the key factors responsible for mucilage’s specific functional potential, while exploring important potential food processing and packaging applications.

## 1. Introduction

Many researchers and food producers are actively exploring alternative ways to improve food quality and safety while minimising the environmental impact. Identifying and investigating novel biopolymers designated as meeting specific processing and environmental requirements in the food industry has recently gained considerable attention [1,2]. It has become essential to find alternative functional biopolymers that address this current global move towards using more sustainably sourced, natural polymers [1,3,4,5].

Although some well-known functional biopolymers, such as pectin, alginate and carrageenan, have been identified, biopolymers that are produced in large quantities, easily sourced and associated with relatively low input costs are a growing area of focus in the research [6]. Mucilage, sourced from the cladodes of the cactus pear, is one such biopolymer that is rapidly gaining attention for its desirable functional properties, which can be utilised to meet current industry demands [3,7,8,9,10]. However, as with all naturally sourced polymers, specific factors have been shown to influence the functional behaviour of mucilage, such as variations in mucilage’s structural composition and the influence of various extraction methods used to obtain the mucilage. These factors should be considered as highly important when evaluating mucilage as a functional biopolymer and ultimately assessing its applications and commercial viability, with a holistic view often lacking in research [11,12,13,14,15,16,17]. Furthermore, it is important to note that the investigation of mucilage has further been promoted from a food, health and medicinal perspective, highlighting its human consumption benefits and safety aspects [6].

The cactus pear, specifically *Opuntia ficus-indica*, the source of mucilage, is a succulent plant that can survive under extreme growing conditions, specifically prolonged drought and extreme heat. Additionally, it has adapted to grow in poor soil conditions, requiring little intervention regarding fertilisation and crop management [6,18]. The plant’s excellent adaptability and survival in times of drought are largely due to mucilage production. Mucilage is responsible for binding and storing water in the plant leaves (called ‘cladodes’) [6]. Consequently, the inherent nature of the mucilage polymer is associated with its excellent water-holding potential. It has, therefore, been evaluated by multiple researchers for its rheology-altering properties when introduced into a solution [18,19,20].

Mucilage, extracted from the cladodes of *Opuntia ficus-indica*, is considered a highly flexible heteropolysaccharide with a high molecular weight [11]. Its primary structure is said to comprise a linear core chain of repeating D-galacturonic acid and L-rhamnose, together with many neutral sugar side chains [15,21,22]. As mucilage has been considered to display polyelectrolyte properties, factors that have been shown to influence other charged biopolymers, such as pectin and alginate, could also be considered to influence the mucilage polymer [19,20,23]. It has generally been well established that mucilage can be associated with two main fractions, differentiated by a charged, pectin-like fraction and a more neutral fraction [15]. The presence of uronic acids associated with the charged fraction of mucilage has specifically been identified, although different authors have reported varying proportions [11,14,15,16]. Various factors account for the differences in the chemical composition of mucilage. One main factor is the methods used to extract mucilage from the cactus pear [12,13,14,21,23].

As mucilage powder is not available commercially, like other well-established biopolymers, authors have evaluated and reported on various mucilage extraction methods. It has been seen that the extraction method selection can directly impact the mucilage yield, highlighting the efficiency of the mucilage extraction process [11,13,15,24]. In addition to the extraction efficacy, the chemical composition and nature of the different mucilage fractions can also be influenced by the extraction method employed, having a consequential impact on the functional properties of the mucilage [11,15,16]. Both of these factors have been identified as directly influencing the rheological behaviour of mucilage solutions [11,13,14,15]. 

Due to mucilage’s structural conformation and inherent water-storing capacity, it has been researched for its viscosity-enhancing potential [19,20,23]. Authors have reported increases in solution viscosity, accounted primarily to the physical entanglement of mucilage’s polymer chains in a solution [11]. Several prominent factors have been shown to alter the rheological properties of mucilage solutions further. These include the alteration of the pH of a solution and the incorporation of cross-linkers, such as calcium and magnesium [19,20,23].

More recently, environmental concerns over the use of non-biodegradable plastic packaging in the food industry have further prompted the investigation of functional biopolymers that display a potential to be used in developing biofilms [25,26,27]. The mucilage from *Opuntia ficus-indica* has specifically been an area of focus in this regard. Research has shown that mucilage has an ever-increasing application in the development of biofilms as a natural packaging [3,8,9,25,28,29]. 

Mucilage has primarily been explored as a polymer in biofilm formation, either as homopolymeric biofilms or in combination with other biomaterials. These films can further be differentiated by their moisture content, with ‘dry’ or low-moisture biofilms being the focus area of many authors [3,30]. Changes in pH, polymer concentration and the presence of a cross-linker have further been shown to directly impact the physical– mechanical properties of these biofilms. A biofilm’s physical–mechanical properties are often considered key when evaluating its packaging development potential [3]. Although mucilage biofilm development has proved successful, variations in the development procedures and the consequential properties displayed by the biofilms have been reported on by different authors. Therefore, it is important to consider and identify the key aspects of the successful development of mucilage biofilms in order to consider them a viable natural packing solution for future exploration [3,9,10,31]. 

Overall, many factors have been shown to influence the functional behaviour of mucilage. These factors provide practical insight into a polymer’s functional potential and, ultimately, its rheological properties and biofilm formation potential [19,20,32,33]. Considering consumer demands, environmental concerns and the high costs associated with commercially available biopolymers, researchers and food manufacturers have focused on identifying and investigating novel biopolymers, such as cactus pear mucilage [9,25]. Mucilage has been shown to display functional properties that can be utilised to address the food industry’s current needs. 

However, with the abundant research often showing considerable variations in findings, a comparative overview of the factors influencing mucilage’s rheological behaviour and biofilm development potential is still lacking. 

Thus, this review article focuses on investigating the specific factors shown to influence the functional behaviour of mucilage sourced from the cactus pear (*Opuntia* spp.), when used as a rheology-altering polymer in a solution and in the application of ‘dry’ biofilms, to provide an accurate, reliable and insightful holistic view of mucilage as a novel functional biopolymer.

## 2. Mucilage Chemical Structure

In general, mucilage is considered a highly flexible heteropolysaccharide with a high molecular weight. Multiple researchers have studied the chemical composition of *Opuntia* spp. mucilage, and have often reported considerable variations therein [11,21,34]. Although variations in the sugar composition and consequential molecular weight have been reported, mucilage molecules are considered to be quite large. This long-chain polymer is represented by varying proportions of D-galacturonic acid and neutral sugars of L-arabinose, D-galactose, L-rhamnose and D-xylose [11,21,22]. The primary structure is said to comprise a linear core chain of repeating (1→4) D-galacturonic acid and (1→2) L-rhamnose.

**Figure 1:**
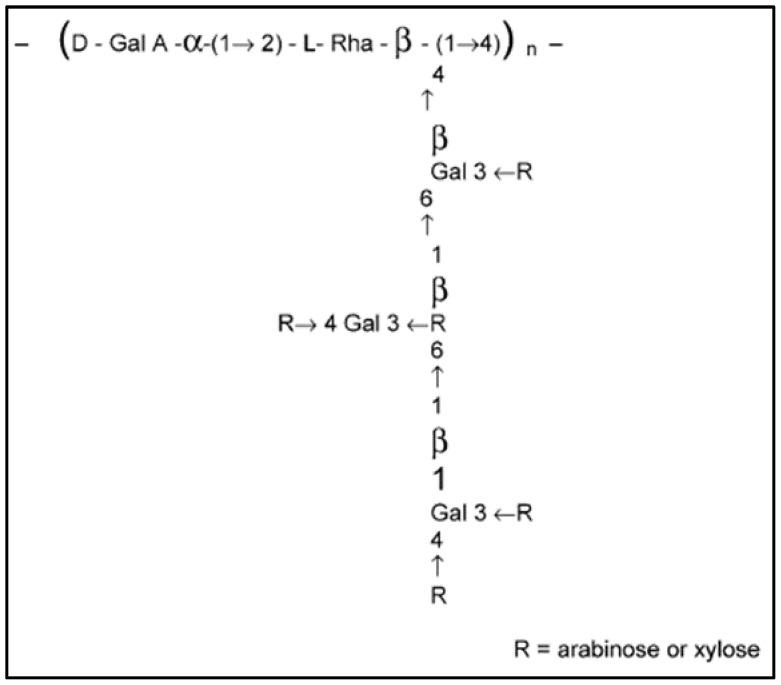
A proposed schematic representation of the mucilage structure of *Opuntia ficus-indica* displaying the charged main linear chain of D-galacturonic acid and L-rhamnose units together with side chains attached to the L-rhamnose residues [21].

Additionally, the mucilage polymer is associated with many side chains of D-galactose attached to L-rhamnose residues. These complex peripheral chains have the potential to comprise various types of sugars. The galactose side chains can further branch into either arabinose or xylose (Figure 1). Native mucilage’s chemical composition can vary considerably, depending on the efficacy and type of extraction method used [12,13,14,21,23]. 

### 2.1. Mucilage Fractions

Investigations of mucilage extracted from cactus pear cladodes have generally shown two prominent, water-soluble fractions present in native mucilage. The first fraction is a pectin-like molecule displaying gelling properties, and the second fraction does not display gel-forming properties [15]. The chemical composition of these two main native mucilage fractions has been shown to differ from one another [23]. The different fractions present within native mucilage extract have been further investigated, and the general conclusions are that native mucilage is represented by both a gelling, pectin-like fraction and a more neutral fraction showing a decreased ability to interact with cross-linkers to form a gel, considered the pure mucilage fraction. Multiple similarities have been observed regarding the chemical composition of these two fractions [15].

In general, the two fractions of native mucilage extract display a similar sugar composition; however, there are differences concerning the percentages of the sugars between the pectin-like and pure mucilage fractions associated with the whole cladodes of *Opuntia* spp. [11]. The differences in sugar percentages are of importance, specifically regarding the content of the charged sugars, such as uronic acid [11,15]. Approximately 20% charged sugars can be present in cladodes, and >50% charged sugars in part of the peel (Table 1). The charged sugars have been identified as galacturonic acid, but the presence of glucuronic acid has also been reported [11,21]. The pectin-like polymer fraction contains a noteworthy greater amount of galacturonic acid compared to the pure mucilage fraction [15]. Uronic acids are important in terms of a polymer’s viscosity-enhancement potential, as their carboxyl groups are able to interact with water molecules, charged metal ions and changes in pH [14].

Considering the proposed chemical structure of mucilage (Figure 1), high amounts of L-arabinose could be expected, as observed in Table 1. Mucilage with higher quantities of arabinose is likely to result in higher viscosities when in an aqueous medium, than that of mucilage with lower amounts of arabinose [14]. Majdoub et al. [11] suggested the high molecular weight of mucilage could be a result of branching at the rhamnose sugars, promoting a random coil conformation of the mucilage polymer in a solution. This is evident when considering the higher levels of neutral sugars (Table 1). 

The unique chemical composition and structural orientation of mucilage are, therefore, likely to result in the formation of highly viscous solutions [14]. In addition to the two main fractions associated with native mucilage, proteins have also been associated with native mucilage. These proteins have been suggested to display decreased water solubility and lower molecular weight compared to pure mucilage fractions [11,15].

Lastly, the high molecular weight, pectin-like polymer fraction will likely display a low degree of esterification, as <50% of their carboxyl groups will be methylated. This pectin-like fraction present in native mucilage is represented by a charged chemical structure, with the potential to interact with divalent cations and respond to changes in pH [11,15,16,35]. 

It is important to recognise the different fractions present in native mucilage as variations in their sugar composition (Table 1). Although the two polymeric fractions associated with native mucilage are not chemically associated, they share a similar composition of neutral sugar residues (Table 1). Specifically, the pectin-like fractions have been linked to higher amounts of uronic acids, with the pure mucilage fractions displaying increased amounts of neutral sugars. The composition of native mucilage will, therefore, greatly determine its observed functional behaviour, consequently influencing its applications [15,16]. Differences in the extraction processes employed by different authors have also been shown to directly affect the sugar composition of the two fractions, as shown in Table 1 [11,12,14,15]. Although similar sugar compositions have been reported for various cultivars of *Opuntia ficus-indica*, differences in the ratios of sugars among different cultivars have been reported, which have a consequential impact on their functional properties [36]. 

### 2.2. Mineral Composition of Mucilage

Besides sugars, mucilage contains essential minerals, carbohydrates and dietary fibre [13,14]. The mineral composition includes calcium (Ca), potassium (K), magnesium (Mg), phosphate (P), zinc (Zn), iron (Fe) and sodium (Na), as well as other minerals in lower quantities. Calcium has been reported to be amongst the most abundant minerals, although significant levels of K and Mg have also been reported [13,19,22,37]. Using X-ray diffraction, Madera-Santana et al. [34] identified crystalline structures displaying the typical characteristics of calcium salts in the mucilage extracted from cladodes. These results agree with those of Contreas-Padilla et al. [37], who confirmed the presence of calcium and calcium oxalate in Opuntia ficus-indica using atomic absorption spectroscopy. For 40–135-day-old cladodes, calcium levels of 17.90–30.70 mg/g dry mass were reported, and increasing calcium levels were also expected for older cladodes. 

## 3. Extraction Methods

The different extraction methods used to extract mucilage have been shown to significantly affect the sugar composition of the native extract (NS), the composition of the different fractions and, ultimately, the functional properties displayed by the mucilage [11,15,16]. Understanding the different extraction methods considered by different researchers and identifying their key differences, as displayed in Table 2. are the purpose of Table 2.

Monrroy et al. [13] evaluated the efficacy of different natural extraction methods to determine mucilage’s viability as a functional food ingredient. The authors investigated extraction methods that employ hydration, agitation and a combination thereof for both dried and fresh cladodes. Hydration extraction was tested with and without thermal treatment on both fresh and dried cladodes. The cladode samples were prepared by peeling, cutting into pieces, crushing and homogenising by suspending 100 mg extract in 5 mL water. For the dried samples, the cladode extract was dried at 60 °C for 48 h before homogenising. The samples were then placed into a water bath at 44–86 °C for 54–96 min, followed by filtration. The mucilage was precipitated from the fresh and dried samples using 45 mL and 15 mL ethanol, respectively, and then oven-dried at 60 °C. A similar protocol was followed for non-thermal hydration extraction, except the sample was allowed to rest for 24 h at room temperature and the heat treatment step was eliminated. The solution was then filtered, and mucilage was precipitated by adding 15 mL ethanol and oven-drying at 60 °C. The extraction through agitation followed a similar protocol for sample preparation as described above, and after homogenisation, the sample was stirred using a magnetic stirrer for 30 min, followed by filtration and precipitation with 15 mL ethanol for 30 min and then dried at 60 °C. Lastly, the combination extraction subjected the samples to 24 h hydration, followed by 30 min of stirring, filtration and mucilage precipitation with ethanol. Considering non-thermal extraction methods for O. *cochenillifera* cladodes, Monrroy et al. [13] reported agitation extraction to be less effective than hydration extraction, and little difference with the combination of agitation and hydration extraction methods. Agitation resulted in about a 26% mucilage yield extraction and hydration about 31%. These results were for dried cladode extraction.

Felkai-Haddache et al. [24] described a process that used the cladodes of *Opuntia ficus-indica* where the outer epidermis was removed from the cladodes, macerated with a domestic mill and then chilled till it reached 4 °C. This extract was then microwaved at 700 W for 5 min and 15 s for optimal mucilage extraction. The optimal amount of water to be added was four times the quantity of the raw material used. The authors also reported that pH plays a significant role in mucilage yield. At pH 11, an optimal amount of mucilage yield was reported because of the high degree of dissociation of the acid groups and the high degree of solubility of the mucilage and, thus, water extraction. After microwaving, the extracted mucilage was cooled (4 °C), filtered to remove the pulp and centrifuged at 4000× *g* for 15 min. The filtrate was precipitated with ethanol (95%, *v*/*v*) at 4 °C overnight. The precipitate was also washed with ethanol (75%, *v*/*v*) and then subjected to lyophilisation at −55 °C for 12 h. Felkai-Haddache et al. [24] reported an optimal mucilage recovery of 25.6%.

In a procedure patented by Du Toit and De Wit [17], an extraction process for mucilage was described, which also used a microwave-heating step. The extraction process requires the cladodes first to be peeled, removing all of the hard outer layers and fibrous material, with only the ‘light green slimy inside’ remaining, which is then sliced into manageable sizes and placed into a microwave oven at maximum power for 4 min (or until the cladode pieces are cooked soft). The cladode pieces are macerated by mincing or cutting without the addition of water. The authors used a juicing apparatus typically found in a household kitchen. Lastly, the macerated mucilage pulp is centrifuged for 15 min at 8000 rpm, maintaining a temperature of 4 °C to separate the solids from the mucilage. There was no reference to an ethanol precipitation step. Mucilage yields of ~39–62% per weight of extracted pulp were obtained, while percentages ranging between 10 and 17% per cladode weight were found.

Majdoub et al. [11] used cladodes (6–12 months old) that were shredded and blended. The mixture was then subjected to degreasing with petroleum ether, washing with deionised water, filtering out the solid components, centrifuging and then filtering again. The supernatant was separated according to different molecular weights using ultrafiltration into high and low molecular weight components. The authors suggested that the presence of proteins in the original sample interacted with the polysaccharides and thus affected the purification results [11]. There was no report of the inclusion of an ethanol precipitation step.

**Figure 2:**
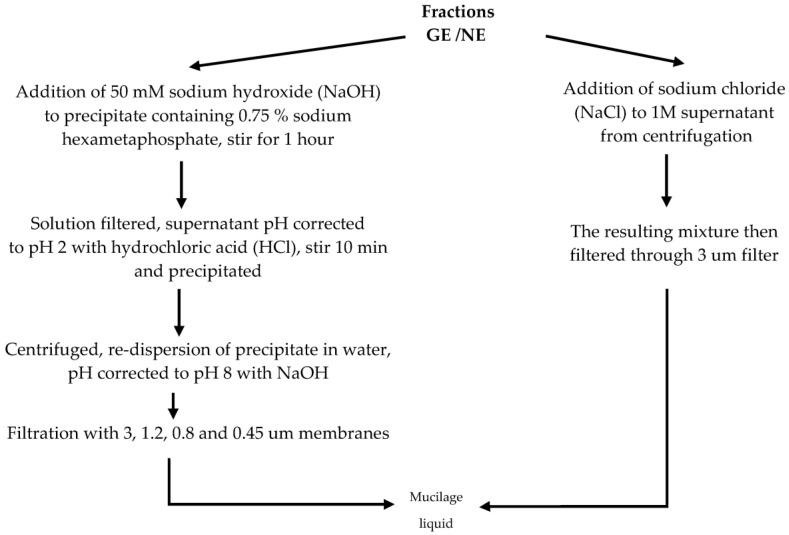
Differences in methods proposed by Goycoolea and Cárdenas [15] to extract and isolate both the gelling fraction (GE) and non-gelling (NE) mucilage/pectin fraction from *Opuntia* spp. cladodes.

Goycoolea and Cárdenas [15] reported on an extraction procedure distinguishing two main mucilage fractions: gelling (GE) pectin and a non-gelling (NE) mucilage fraction. For the extraction of both fractions, cladodes were diced and heated for 20 min at 85 °C, then neutralised with sodium hydroxide (NaOH), liquidised and then centrifuged. The resulting precipitate was considered the GE fraction, and the supernatant the NE fraction. The precipitate and supernatant were subjected to the extraction protocols represented in Figure 2. After these separate procedures were completed (Figure 2), both fractions were again precipitated with 50% *v*/*v* ethanol, again centrifuged, then washed with ethanol/water mixtures (70, 80, 90, 95 and 100% *v*/*v*) and then dried at room temperature.

## 4. Functional Properties Associated with Mucilage

Various researchers have explored multiple procedures detailing the extraction and purification of mucilage [13,14,21]. Investigations of the rheological and physico-chemical behaviour of native extracts from cladodes have highlighted the important factors affecting the implementation of mucilage as a functional food ingredient [11,32,38]. Certain dominant factors have been identified that influence the functional behaviour of mucilage in a solution. Specifically, the polymer concentration, pH and presence of a cross-linker in a solution considerably alter mucilage’s functional behaviour [19,20,23].

### 4.1. Rheology-Altering Properties of Mucilage

As a result of mucilage’s pseudoplastic nature and unique structural conformation aspects, it has an excellent water-holding capacity. The viscosity-enhancement properties of mucilage in an aqueous solution have been directly linked to the physical entanglements between the polymer chains [11]. Specifically, Medina-Torres [23] found similar viscosity-altering capabilities of a 10% mucilage solution to that of 3% xanthan solutions in aqueous mediums. Monrroy et al. [13] also compared mucilage’s thickening ability to that of commercially available hydrocolloids. The authors noted similarities between the ability of mucilage and gum arabic to form low-viscosity solutions [13].

The molecular weight, chain flexibility and surface charge were found to influence the interfacial activity of mucilage (i.e., mucilage’s ability to mix with water) [39]. In general, an increase in mucilage concentration, together with high chain flexibility and the presence of uronic acid along its polymer backbone, can promote the adsorption of mucilage onto the liquid phase [11,39]. Water absorption by the polymer is directly influenced by the amount of active water-binding sites, which are affected by the physico-chemical, topological and structural parameters [39].

Generally, viscosity increases by the dispersion of mucilage into aqueous solutions, which has been accounted for by the negatively charged nature of the polymer. These similarly charged, long, flexible mucilage molecules repel themselves in a solution, resulting in the uncoiling and stretching out of the molecule. This is often referred to as electrostatic repulsion. This ‘stretching’ of a molecule is predominantly responsible for viscosity increases in a solution [11]. A mucilage polymer’s thickening ability is further aided by the intermolecular interactions of its polymer side-chain groups via hydrophobic interactions or hydrogen bonding. These viscous solutions formed by mucilage have been shown by Medina-Torres et al. [23] to display non-Newtonian shear-thinning behaviour. This behaviour of increased pseudoplasticity was noted for solutions with increasing amounts of mucilage. Majdoub et al. [11] also confirmed this behaviour by explaining that the zero-shear viscosity value increases with an increase in the concentration of the polymer.

Du Toit et al. [33] investigated the rheological behaviour of mucilage from *Opuntia* spp. Non-Newtonian, pseudoplastic flow properties, indicative of a shear-thinning effect of mucilage, were observed by the authors [33]. They further reported that the viscosity of mucilage solutions are influenced by changes in pH and the presence of charged ions, specifically CaCl_2_ and FeCl_3_, highlighting the functional nature of mucilage [33].

The non-Newtonian, pseudoplastic flow behaviour was further reported on by a more recent study conducted by van Rooyen et al. [20]. The authors found that increasing the concentration of mucilage in an aqueous solution resulted in the increased pseudoplastic flow behaviour of the mucilage solution. Interestingly, the authors found that mucilage possesses an overall poorer viscosity-enhancement potential compared to other commercially available biopolymers, such as pectin and alginate, at all the polymer concentrations investigated [20]. Although mucilage has the ability to alter the rheology of a solution, certain factors have been shown to directly influence mucilage’s functional behaviour in a solution. Other than polymer concentration, the pH of a solution and the presence of a cross-liker must be considered key influencing factors when evaluating the rheological behaviour of mucilage in a solution [19,20,33,38].

#### Factors Influencing the Rheology of a Mucilage Solution

Due to the presence of charged sugars, the viscosity of a mucilage solution has been shown to be influenced by the presence of cations, specifically calcium [11,20]. Since the native extract from cactus pear cladodes has been shown to comprise various gelling and non-gelling fractions, the efficacy of the extraction and purification methods used have the potential to greatly influence the functionality displayed by the extracted biopolymer from cactus pear cladodes. Therefore, diverse and often contradictory findings have been reported on the potential of cactus pear (*Opuntia* spp.) extracts to form a gel or display gel-like properties [11,14,15,21].

It has been suggested that mucilage displays elastic properties but is unable to form a gel, regardless of the ionic strength of a solution [11,23]. Majdoub et al. [11] reported on a native polysaccharide extract from cladodes that displayed a slight polyelectrolyte effect, as the molecule’s confirmation and viscosity were shown to be influenced by the addition of cations. The authors reported that monovalent cations have less of an effect on viscosity than divalent cations, such as calcium. However, even with the addition of calcium, only slight changes in viscosity were observed, indicative of the limited extension of the polymer in an aqueous solution, which accounted for the low proportion of uronic acids present in the polymer investigated [11]. For mucilage, however, a loss in viscosity is related to a high degree of chain flexibility of the polymer and a low occurrence of changed uronic acid residues. This loss of viscosity with the addition of salt has also been referred to as the salting-out effect. For mucilage, physical gel formation is unable to be occur as expected, as the formation of “inactive loops” prevents the formation of intermolecular junctions between polymers that are required to form a 3D gel network. However, solvent conditions will influence a polymer’s chain–chain interactions and configuration [11]. The rheological properties of ionic gels depend on the concentration of cations and the presence of uronic acid in the mucilage. Thus, low uronic acid and few cations have little effect on the viscosity of mucilage in an aqueous system.

However, reports of extracts from cactus pears containing higher contents of charged sugars, displaying increased gel-like properties by the addition of cations, such as Ca^2+^, have also been reported. The pectin-like fraction present in native mucilage was suggested to be the reason for these observed gel-like properties associated with native mucilage [15,19,20].

The presence of charged sugars has been reported as a significant parameter with which to modulate the structuring capabilities of a mucilage polymer, resulting in mucilage displaying pectin-like behaviour with consequential gel-like properties if certain parameters are met [39]. It has, therefore, also been noted that native mucilage has the potential to be influenced by changes in solution pH. As charged sugars have been associated with native mucilage’s chemical structure, changes in pH have been shown to consequently alter the rheological properties of native mucilage. Instances of native mucilage solutions displaying increased gel-like behaviour have been reported under acidic conditions, generally related to a type of acid gelation displayed by native mucilage [19].

### 4.2. Mucilage in the Development of Biofilms

#### 4.2.1. Homopolymeric Mucilage Biofilms

Cactus pear mucilage has also been considered as an ingredient for biofilms’ development. Research on mucilage biofilms and edible coatings has increased considerably, but research remains relativity limited compared to that on other biopolymers, such as pectin and alginate. Biofilm characteristics are generally reported in one of two states, either as ‘dried’ or ‘wet’ [25,40,41]. In the case of mucilage biofilms, authors have generally reported on the films when they are in a dried state, as mucilage has been shown to have a poor gelling capacity with cations typically. The mechanical properties of biofilms are of great importance, as these parameters are directly related to their chemical structure and potential for commercial application [41,42,43]. However, as with most dried polysaccharide-based films, the addition of a plasticiser has been set as the standard for developing mucilage-based biopolymer films. ‘Dried’ mucilage films have generally displayed brittle and fragile properties if no plasticiser is used in their development. Adding a plasticiser, usually glycerol, allows for more flexible mucilage films that display adequate handling and mechanical properties [44,45,46]. In research conducted by Gheribi et al. [41], the authors specifically found that using glycerol as a plasticiser at a 40% *w*/*w* in the development of mucilage (*Opuntia ficus-indica*) films produced films with superior elongation at break (*%E*) values, together with adequate film tensile strength. It has been suggested that natural plasticisers, such as glycerol, are essential in developing biopolymer films that show adequate film-forming properties if intended to be used as natural packaging [9,10,41]. As with other commercially available biopolymers used in the development of biofilms, mucilage films have also been developed both with and without the addition of cross-linking agents and at varying pHs. Limited comparative work is available on these factors’ influence on mucilage biofilm properties. More recently, publications have explored these factors in more detail [9,10,31,43].

Espino-Díaz et al. [31] were one of the first authors to investigate biofilm formation using mucilage from *Opuntia ficus-indica*. The authors developed these mucilage biofilms together with 50% glycerol, which was added based on the weight (*w*/*w*) of the mucilage used in the films [31]. These mucilage films were produced at different pH ranges (3, 4, 5, 6, 7 and 8), with and without the addition of calcium (in the form of CaCl_2_) as a cross-linker. The pH was adjusted using hydrochloric acid or sodium hydroxide to achieve the desired pH. At the native pH, without the addition of calcium, the films showed the highest TS. Alternatively, the films exposed to calcium displayed enhanced %E and enhanced changes in pH. An important finding regarding the adhesion of the films was further highlighted by the authors; improved film strength and handling was achieved at a higher pH range (pH 5–8) because at a low pH (pH 3), the films were difficult to work with due to the high adhesion potential and elasticity [31].

It has been observed that cross-linked films typically result in a less compact structure due to the intermolecular linking with calcium, resulting in the reduction in and tying of the polymer chains. However, without the addition of calcium, there is a higher chain flexibility, allowing for the formation of more compact films, as there is more contact surface among the molecules [31]. This was supported by Livney et al. [47], who suggested that greater conformation flexibility was possible for open structures than for intermolecular cross-linked structures. Practically, films formed without the addition of calcium allow for reduced permeability as a more organised and compact 3D structure network is formed, retarding movement across the film when compared to films with calcium incorporated into them [31]. Importantly, if mucilage is at a low pH of 4, it is positively charged and may result in reduced intermolecular bonding with calcium [31]. Some authors have reported increased film TS values associated with polymers of increased molecular weight [31,48].

Lira-Vargas et al. [43] are some of the few researchers who have also reported on the potential of using mucilage to form biopolymer films. These authors investigated the non-gelling mucilage fraction extracted from the cladodes of *Opuntia ficus-indica*. Films were formed by drying without the addition of a cross-linker. The films displayed an inconsistent surface morphology, with a lumpy and granular texture. These mucilage biopolymer films resulted in a TS average of 0.49 MPa, similar to that reported by Espino- Díaz et al. [31]. More recent studies have also shown that mucilage can be successfully used in the development of single-polymer films [8,49].

Van Rooyen et al. [10] reported on the mechanical properties of mucilage biofilms. The polymer concentration and changes in pH were shown to have a considerable influence on the biofilm’s mechanical properties [10]. It was found that mucilage films developed at a low polymer concentration of 2.5% (*w*/*w*) were unable to produce films displaying satisfactory mechanical properties. However, increasing the polymer concentration of the mucilage in the films to 5% (*w*/*w*) and 7.5% (*w*/*w*) resulted in mucilage films displaying adequate mechanical properties [10]. Furthermore, when decreasing the pH of mucilage films to pH 3–3.5, a noticeable increase in the films’ elasticity (when considering the elongation at break % measurements) was reported. This trend of increased film tensile strength at a pH 3–3.5 was ascribed to a type of acid by the authors, indicating the functional nature of mucilage [10].

In addition to changes in pH, Brandon van Rooyen et al. [9] further showed that calcium, in the form of calcium chloride, could alter the physical properties of mucilage biofilms. The authors specifically reported that calcium reduces elasticity and increases the strength of mucilage biofilms [9].

#### 4.2.2. Composite/Blended Mucilage Biofilms

Although the benefits of composite biopolymer films have been well established in the literature, limited research is available on mucilage’s interaction with other commercial polymers. It has been suggested that compatible chemical synergetic interactions between ingredients displaying similar mechanical profiles could result in enhanced film mechanical properties. That would be essential for developing low-cost, biodegradable packaging [3,8,29,40,50].

In the recent work of Van Rooyen et al. [3], the authors investigated a blend of mucilage in combination with pectin and mucilage in combination with alginate biofilms. The influence of calcium as a cross-linker was also considered. It was found that, overall, pectin and mucilage displayed a synergist interaction, producing biofilms of increased strength. In contrast, alginate and mucilage showed a poor interaction with each other, reducing film strength. Polymer compatibility accounted for these differences, with pectin and mucilage showing a positive correlation [3].

Andreuccetti et al. [28] investigated the influence that mucilage (*Opuntia ficus-indica*) would have in combination with starch, with glycerol as a plasticiser, to improve film processability, functionality and certain mechanical properties. Composite rice starch and mucilage films produced adequate film mechanical properties, reporting a TS range of 2.80–3.96 MPa and *%E* of 12.07–20.63%. The authors suggested that combining the various biomaterials in the films allowed for a synergistic interaction between starch, mucilage and glycerol. These synergistic interactions resulted in increases in the films’ *%E* values, coinciding with decreases in the films’ TS values [15].

In another study, Sandoval et al. [8] investigated the effect that various cultivars’ mucilage (*Opuntia ficus-indica*) would have on pectin-based film properties for developing biodegradable mucilage films. The authors suggested that determining a film’s puncture tests would determine the ability of the film to maintain structural integrity and offer protection to the food product, as it would directly evaluate the film’s mechanical strength. It was reported that different *Opuntia* spp. cultivars’ mucilage influenced the mechanical properties of composite films to different degrees. The authors also found that decreases in the intermolecular association between polymers in the film matrix consequently resulted in the film’s displaying increased flexibility/elasticity and, consequently, decreased film strength. The low polymer concentration used in the development of the films was also suggested to be a reason for this occurrence [8].

Luna-Sosa et al. [50] also investigated the influence mucilage would have on the properties of composite pectin films together with glycerol. The films were developed using the ‘casting’ method and then dried prior to investigation. The addition of mucilage was shown to directly influence the single-polymer pectin films’ morphological and mechanical properties. The authors suggested that mucilage interfered with the pectin film matrix, resulting in less compact film morphological structures. These composite films further showed reduced mechanical properties for both TS and *%E* in comparison to the single-polymer pectin films [50].

Scognamiglio et al. [51] comparatively investigated the tensile tests and microstructures of composite mucilage films developed with thermoplastic starch and high amounts of glycerol. Mucilage was shown to negatively influence the films’ TS, showing a more than 50% reduction in film strength in comparison to the single-polymer thermoplastic starch films. A TS range of 0.68–1.64 MPa was reported for composite mucilage films. The films’ *%E* values, however, were increased by the addition of mucilage, interestingly, in some instances more than 50%, when compared to the single-polymer thermoplastic starch films. The authors further suggested that although high amounts of Ca and Mg were associated with mucilage, these did not seem to alter the mechanical performance of the films, suggesting that they were not bioavailable to alter the functional behaviour of the films.

### 4.3. Limitation and Future Perspectives

Variations in extraction methods, coupled with the varying physiological and environmental growth conditions of *Opuntia* spp. have resulted in considerable variation in the functional properties displayed by mucilage. In addition, specific factors have been identified as further altering the functional behaviour of mucilage in a solution and in the development of biofilms, limiting the consistency and expected outcomes of mucilage functional behaviour and applications. The presence of a cross-linker, polymer concentration and alteration in pH have specifically been identified and discussed in this review, with variations observed between different authors [19,20,23].

With regards to mucilage biofilm development, only more recently have specific publications been produced that address mucilage use in the development of biodegradable packaging in the food industry [3]. However, the limited work available on mucilage used in composite film development often shows inconsistent and contradictory findings. Nevertheless, a general consensus indicates that mucilage shows an undoubtable potential for use in the development of composite biofilms to be used as biodegradable packaging in the food industry [8,50].

As knowledge of the interactions between mucilage and other well-established polymers remains limited or non-existent, further investigations of the possibility of various cultivars’ mucilage being used in the development of biopolymer films must be strongly considered. Understanding mucilage’s functional behaviour relative to that of well-established functional polymers would further prove essential when holistically evaluating mucilage as a functional food ingredient for various applications. These findings could be used to improve the physical properties of packaging further and reduce the gap between low-cost, biodegradable packaging and single-use, petroleum-based packaging.

## 5. Conclusions

Consumer demands and environmental concerns have been a driving force behind the identification and investigation of novel natural biopolymers, such as cactus pear mucilage. Mucilage has specifically been considered for its unique and desirable functional properties that have been used in an attempt to address the current needs of the food industry. Compared to other commercially available polymers, limited research is available on mucilage’s overall functional potential as a natural biopolymer and its commercial feasibility. Specifically, the functional potential of mucilage has found applications from a rheological and biofilm development perspective. It has, however, been seen that mucilage displays a functional potential that can be manipulated by a variety of factors, such as pH, a cross-linker and further influenced by polymer concentration. Variations amongst the different extraction procedures have also been identified as a critical factor for determining the functional behaviour of the resultant mucilage. It is suggested that mucilage’s functional properties and applications require additional investigation. A better understanding of the functional behaviour of mucilage will aid in furthering and developing optimal extraction procedures. Although mucilage’s rheological behaviour and biofilm development formation have been investigated more recently, certain aspects are still lacking from a research and application perspective. Therefore, further investigation of mucilage will prove critical for making future recommendations on mucilage’s functional potential, especially if certain industry requirements and applications must be achieved.

## Figures and Tables

**Table 1:** Sugar composition of the pectin-like and mucilage fractions associated with *Opuntia ficus-indica* cladodes (% in weight).

Sugars Investigated **	Pectin Fraction (A)	Pectin Fraction (B)	Mucilage Fraction (A)	Mucilage Fraction (C)	Mucilage Fraction (D)
Uronic acid *	56.30	85.40	11.00	19.4	13.91
Arabinose	5.60	6.00	17.93	33.10	35.36
Galactose	6.50	7.00	20.99	20.30	27.26
Rhamnose	0.50	0.60	1.75	6.90	1.93
Xylose	0.90	1.00	3.06	18.7	16.32
Research performed by:					
(A) Goycoolea and Cárdenas [15]; (B) Cárdenas et al. [16]; (C) Majdoub et al. [11]; (D) Rodriguez-González et al. [14].					

* Galacturonic + Glucuronic acid. ** Other component may have been present and reported on by the various authors.

**Table 2:** Summary of key extraction method steps described in various publications by different authors together with their expected mucilage yields. representation of the different extraction methods investigated by various authors and their extraction efficacy.

Publication	Heating/Microwave Treatment	Maceration/Blending/Milling	Filtration	Centrifugation	Ethanol Extraction	Moisture Removal	Mucilage Yield
Monrroy et al. [13]	√	√	√	–	√	Oven-dried	~24–31%
Felkai-Haddache et al. [24]	√	√	√ Cheesecloth	√	√	Freeze-drying	~6.82–25.56%
Du Toit and De Wit PA153178P [17]	√	√	–	√	–	Freeze-drying	~39–62% Native Mucilage ***
Majdoub et al. [11]	–	√	Filtration + Ultrafiltration	√	–	Freeze-drying	Separate Fractions **
A review: Goycoolea and Cárdenas [15]	√	√	Complex Filtration; pH Adjustment	√	√ Multi-step	* DNS	Separate Fractions **

* DNS—Publication did not specify; ** Separate Fractions—extraction procedure described different mucilage fractions present; *** Native Mucilage—mucilage obtained was not separated according to different fractions.

## Data Availability

Not applicable.
